# Changes in Soluble CD18 in Murine Autoimmune Arthritis and Rheumatoid Arthritis Reflect Disease Establishment and Treatment Response

**DOI:** 10.1371/journal.pone.0148486

**Published:** 2016-02-05

**Authors:** Tue Wenzel Kragstrup, Babak Jalilian, Kresten Krarup Keller, Xianwei Zhang, Julie Kristine Laustsen, Kristian Stengaard-Pedersen, Merete Lund Hetland, Kim Hørslev-Petersen, Peter Junker, Mikkel Østergaard, Ellen-Margrethe Hauge, Malene Hvid, Thomas Vorup-Jensen, Bent Deleuran

**Affiliations:** 1 Department of Biomedicine, Aarhus University, Aarhus, Denmark; 2 Department of Rheumatology, Aarhus University Hospital, Aarhus, Denmark; 3 Copenhagen Center for Arthritis Research, Rigshospitalet, Glostrup, Denmark; 4 Department of Clinical Medicine, University of Copenhagen, Copenhagen, Denmark; 5 University of Southern Denmark, Odense, Denmark; 6 Odense University Hospital, Odense, Denmark; 7 Department of Clinical Medicine, Aarhus University, Aarhus, Denmark; University of Leuven, Rega Institute, BELGIUM

## Abstract

**Introduction:**

In rheumatoid arthritis (RA) immune activation and presence of autoantibodies may precede clinical onset of disease, and joint destruction can progress despite remission. However, the underlying temporal changes of such immune system abnormalities in the inflammatory response during treat-to-target strategies remain poorly understood. We have previously reported low levels of the soluble form of CD18 (sCD18) in plasma from patients with chronic RA and spondyloarthritis. Here, we study the changes of sCD18 before and during treatment of early RA and following arthritis induction in murine models of rheumatoid arthritis.

**Methods:**

The level of sCD18 was analyzed with a time-resolved immunoflourometric assay in 1) plasma from early treatment naïve RA patients during a treat-to-target strategy (the OPERA cohort), 2) plasma from chronic RA patients, 3) serum from SKG and CIA mice following arthritis induction, and 4) supernatants from synovial fluid mononuclear cells (SFMCs) and peripheral blood mononuclear cells (PBMCs) from 6 RA patients cultured with TNFα or adalimumab.

**Results:**

Plasma levels of sCD18 were decreased in chronic RA patients compared with early RA patients and in early RA patients compared with healthy controls. After 12 months of treatment the levels in early RA patients were similar to healthy controls. This normalization of plasma sCD18 levels was more pronounced in patients with very early disease who achieved an early ACR response. Plasma sCD18 levels were associated with radiographic progression. Correspondingly, the serum level of sCD18 was decreased in SKG mice 6 weeks after arthritis induction compared with healthy littermates. The sCD18 levels in both SKG and CIA mice exhibited a biphasic course after arthritis induction with an initial increase above baseline followed by a decline. Shedding of CD18 from RA SFMC and RA PBMC cultures was increased by TNFα and decreased by adalimumab.

**Conclusions:**

The plasma sCD18 levels were altered in patients with RA, in mice with autoimmune arthritis and in cell cultures treated with TNFα and adalimumab. Decreased levels of plasma sCD18 could reflect autoimmunity in transition from early to chronic disease and normalization in response to treatment could reflect autoimmunity in remission.

## Introduction

Rheumatoid arthritis (RA) is characterized by swollen and painful joints caused by immune system abnormalities [1]. However, seropositivity for autoantibodies like rheumatoid factor and anti-citrullinated protein antibodies may precede clinical onset of disease [2], and joint damage can progress despite clinical remission [3]. This indicates, that immune system activation may be present in preclinical RA and in RA in clinical remission. Therefore, early and aggressive suppression of synovitis and overactive immune system pathways are principal goals in current treat-to-target strategies [4]. However, not much is known about the temporal course of immune system activation during disease development and immune system resetting during treatment.

The inflammatory response includes many different components. The family of β2 (CD18) integrins (comprising LFA-1 (CD11a/CD18), complement receptor 3 (CD11b/CD18 or Mac-1), complement receptor 4 (CD11c/CD18 or p150,95), and CD11d/CD18) is central in the inflammatory response and in RA. E.g., LFA-1 permits leukocytes to bind ICAM-1 and migrate to inflammatory foci [5]. Blocking this interaction between β2 integrins and their ligands ameliorates arthritis in both animal models of RA and RA [6–10]. β2 integrin small molecule antagonists are under evaluation for the treatment of other autoimmune diseases.[11] We and others have demonstrated a soluble form of CD18 (sCD18) resulting from sheddase activity [12–18]. Shedding of CD18 is increased during chemotaxis and following stimulation with TNFα [12,16,17], and the sCD18 complexes compete with the cell-expressed CD18 integrins for binding to ICAM-1 [12,19]. The plasma concentration of sCD18 seems to be a result of a balance between production by shedding and depletion by ligand binding, and plasma sCD18 may function as a regulatory factor by limiting leukocyte adhesion. In chronic RA and chronic spondyloarthritis, the plasma levels of sCD18 are decreased and associate inversely with disease activity [12,19].

Here, we study changes in plasma sCD18 levels (1) in patients with early RA before and during a treat-to-target strategy (patients from the OPERA cohort), (2) in chronic RA patients, (3) following arthritis induction in murine models of rheumatoid arthritis (the SKG and CIA models) and (4) in RA synovial fluid mononuclear cell (SFMC) and peripheral blood mononuclear cell (PBMC) cultures.

## Methods

### Patients and healthy controls

Serial plasma samples were obtained from RA patients participating in the OPtimized treatment algorithm in Early RA (OPERA) study (n = 152) (Table 1). The 152 patients used in this study were randomly selected among the 180 patients included in the trial. Detailed study design and outcome measures have been published elsewhere [20,21]. Briefly, the patients were treatment naïve early RA patients with median disease duration before diagnosis of 84 days (IQR 43–132 days). Upon inclusion the patients were randomized to a step-up methotrexate protocol in combination with either adalimumab (Humira, Abbvie, Illinois, USA) or placebo. Patients in either treatment arm received intra-articular glucocorticoid injections when presenting with synovitis as assessed clinically. In this study, we used plasma samples from the initiation of treatment (0 months) and after 3, 6, and 12 months of treatment. Clinical assessments were obtained at baseline (0 months) and after 3, 6, 12, and 24 months of treatment. Conventional radiographs of hands and forefeet were scored according to the Sharp/van der Heijde method at baseline (0 months) and after 6, 12, and 24 months (Table 1). The intraobserver intraclass correlation coefficient for total Sharp score (TSS) change was 0.88 (95% CI 0.72 to 0.95).

**Table 1:** Characteristics of the early treatment naïve RA patients.

	Early RA patients (n = 152)				
	0 months	3 months	6 months	12 months	24 months
**Age (years)**	56 (43–63)	-	-	-	-
**Gender (% female)**	69	-	-	-	-
**Baseline characteristics**					
** Disease duration (days)**	84 (43–130)	-	-	-	-
** RF (% positive)**	71	-	-	-	-
** Anti-CCP (% positive)**	65	-	-	-	-
** Treatment (% placebo)**	50	-	-	-	-
**Disease activity**					
** CRP (mg/L)**	15 (7–40)	7 (7–11)	7 (7–10)	7 (7–8)	7 (7–7)
** Patient global (1–100)**	67 (42–83)	10 (3–29)	14 (3–41)	15 (2–30)	10 (2–29)
** Physician global (1–100)**	56 (43–73)	4 (0–12)	4 (0–11)	2 (0–11)	2 (0–5)
** HAQ (0–3)**	1.1 (0.8–1.8)	0.1 (0–0.6)	0.1 (0–0.6)	0.1 (0–0.5)	0.1 (0–0.5)
** DAS28CRP (0–10)**	5.6 (4.9–6.3)	2.2 (1.8–3.1)	2.4 (1.8–3.0)	2.0 (1.8–2.8)	2.0 (1.8–2.7)
**ACR response (% responders)**					
** ACR20**	-	88	85	86	84
** ACR50**	-	69	73	75	75
** ACR70**	-	54	57	59	59
** ACR90**	-	32	25	34	32
**Radiographic progression (% progressors)**					
** TSS**	-	-	32	41	48
** Erosions**	-	-	27	32	37
** JSN**	-	-	20	23	31

Data are expressed as median with IQR. Months indicate time after inclusion (treatment initiation). RF, rheumatoid factor. Anti-CCP, anti-cyclic citrullinated peptide antibody. CRP, C-reactive protein. HAQ, health assessment questionnaire. DAS28CRP, disease activity score 28 based on C-reactive protein. ACR, American College of Rheumatology improvement score. TSS, total Sharp score. JSN, joint space narrowing.

Plasma from patients with chronic RA was obtained from another study population (n = 30). At the time of inclusion, patients presented with disease flare with at least one swollen joint. Median age was 58.5 years (IQR 44.8–66.3 years) and the percentage of females was 66.7%.No disease activity or prognosis scores or test results were available. A subgroup of this population was randomly selected for use in PBMC and SFMC culture stimulation and inhibition experiments (n = 6).

Plasma was also collected from age- and gender-matched healthy controls (HCs) from the Donor Bank at Aarhus University Hospital (n = 88). Median age was 53 years (IQR 46–61 years) and the percentage of females was 68%. All plasma samples were collected in EDTA tubes and kept at -80°C until use. PBMC and SFMC were isolated by conventional Ficoll-Paque (GE Healthcare) density-gradient centrifugation and cryopreserved at -135°C until time of analysis.

### SKG mice

Serum samples from a total of 45 SKG mice were included from a previous study [22]. Detailed description of animals and arthritis induction has been described previously [22,23]. Briefly, female 11- to 12-week-old SKG mice were equally randomized into five groups with nine mice in each group. Three groups of animals had arthritis induced by intraperitoneal injection of 20 mg mannan (M7504, Sigma-Aldrich, USA), the animals in another group had arthritis induced with intraperitoneal injection of 2 mg zymosan A (Z4250, Sigma-Aldrich USA), and the animals in the last group served as age-matched controls. The arthritis was scored as described by Sakaguchi et al by an observer blinded for group distribution [24]. After 14, 28, and 42 days the mice were anesthetized by inhalation with isoflorane (IsoFlo vet, Abbott Laboratories Ltd. Kent, UK), serum was collected from the orbital sinus, and the mice were sacrificed by cervical dislocation.

### CIA mice

Longitudinal serum samples from a total of 4 female C57BL/6 mice with CIA were included. Mice were 11–12 weeks of age (Taconic, Ry, Denmark) and kept in Scantainers under SPF conditions (21–25°C, 30–60% humidity and 12-hour light/dark cycle). For development of CIA, the mice were immunized with chick collagen II and Complete Freund’s Adjuvant at baseline and after 21 days. Arthritis scores were done in accordance with previously described methods [25]. After 56 days the mice were sacrificed.

### PBMC and SFMC in vitro cultures

SFMC and PBMC were thawed and cultured in culture medium (RPMI medium supplemented with 10% (v/v) FCS, penicillin, streptomycin, and glutamine) at a density of 2×10^6^ cells/ml. Cells were grown in either medium alone or medium supplemented with either TNFα at 40 ng/ml (300-01A, Peprotech) or adalimumab at 5 μg/ml (Humira) for 48 h at 37°C in a humidified incubator 5% (v/v) CO_2_ without changing of medium. After incubation, supernatants were stored at -80°C for later sCD18 analysis.

### Measuring human sCD18

The concentration of sCD18 in human plasma and culture supernatants was carried out with a time resolved immunofluorescence assay (TRIFMA) as described previously [12,26]. In brief, microtiter plates were coated with KIM185 capture antibody (hybridoma cell line CRL-2839, GenScript). Following incubation with plasma diluted 1:10 or supernatants diluted 1:2 the detection of sCD18 was achieved by incubation with biotinylated KIM127 (hybridoma cell line CRL-2838, GenScript) and Eu3+-conjugated streptavidin and read in a fluorescence plate reader. A human plasma sample defined to contain 1,000 mU/ml was used to make a standard curve and two internal controls were used to correct for interassay variations. Buffers contained bovine and human immunoglobulins to block interference from anti-antibodies such as rheumatoid factor in the patient samples [19,26]. Also, signals were read in plates coated with isotype mouse IgG1 monoclonal antibody (M7894, Sigma-Aldrich) with no known reactivity as a negative control.

### Measuring mouse sCD18

The measurement of sCD18 concentration in mouse serum was carried out with a TRIFMA as described previously [18]. Briefly, microtiter plates were coated with a rat anti-mouse CD18 IgG2a capture antibody (clone 313903) at a concentration of 1 μg/ml in PBS pH 7.4 at 4°C overnight. Samples were diluted 1:10 in TBS/Tween/azid assay buffer containing 1 mM CaCl_2_, 1 mM MgCl_2_, and 100 μg/ml heat aggregated human IgG, 100 μg/ml bovine IgG 100 μg/ml rat IgG, and 100 μg/ml mouse IgG and added to the plates. After incubating the plates overnight at 4°C and following three washes with TBS/Tween/azide, a biotinylated rat anti-mouse CD18 detection antibody (clone C71/16) was added at a concentration of 1 μg/ml in TBS/Tween/azid assay buffer with 1 mM CaCl_2_ and 1 mM MgCl_2_ for 1 hour at room temperature. Following three washes, signal was developed by incubation with Eu3+-conjugated streptavidin in TBS/Tween with 25 μM EDTA for 1h at room temperature and read in a fluorescence plate reader. A mouse serum sample defined to contain 1,000 mU/ml was used to make a standard curve. Also, signals were read in plates coated with isotype rat IgG2a monoclonal antibody (11021D, BD biosciences) as a negative control.

### Ethics

All human samples were obtained after informed written consent according to the Declaration of Helsinki. The Danish Data Protection Agency and the Ethics Committee at Region Midt approved the OPERA study (20070008)[20] and the collection of synovial fluid and peripheral blood from chronic RA patients for isolation of plasma, SFMCs and PBMCs (20121329).

The Danish Animal Experiment Inspectorate under the Danish Ministry of Environment and Food approved both the study using SKG mice (2007/561-1317) and the experimental protocol using CIA mice (2009/561-1667).

### Statistics

Patient characteristics were described by the median and interquartile range (IQR). Comparisons of the plasma sCD18 levels between groups of unpaired samples were made using the t-test on log-transformed data. Comparisons of the plasma sCD18 levels between groups of paired samples were made with the paired t-test on log-transformed data. Comparisons of changes in plasma sCD18 levels between ACR non-responders and responders were made using the t-test. Correlation analyses between sCD18 levels and patient clinical scores and test results were performed with the Spearman correlation. Comparisons of the mouse serum sCD18 levels between groups of unpaired samples were made using the t-test on log-transformed data. Cell culture experiments were analyzed with nonparametric statistics. Thus, the Mann-Whitney U test was used for unpaired comparisons and the Wilcoxon signed rank test was used for paired data. A two-tailed p-value below 0.05 was considered significant. Calculations and graphs were made with Stata version 11.1 (StataCorp, College Station, USA) and GraphPad Prism version 5 (GraphPad Software, San Diego, CA).

## Results

### The plasma levels of sCD18 were decreased in established RA and exhibited a biphasic course during a treat-to-target strategy

**Fig 1:**
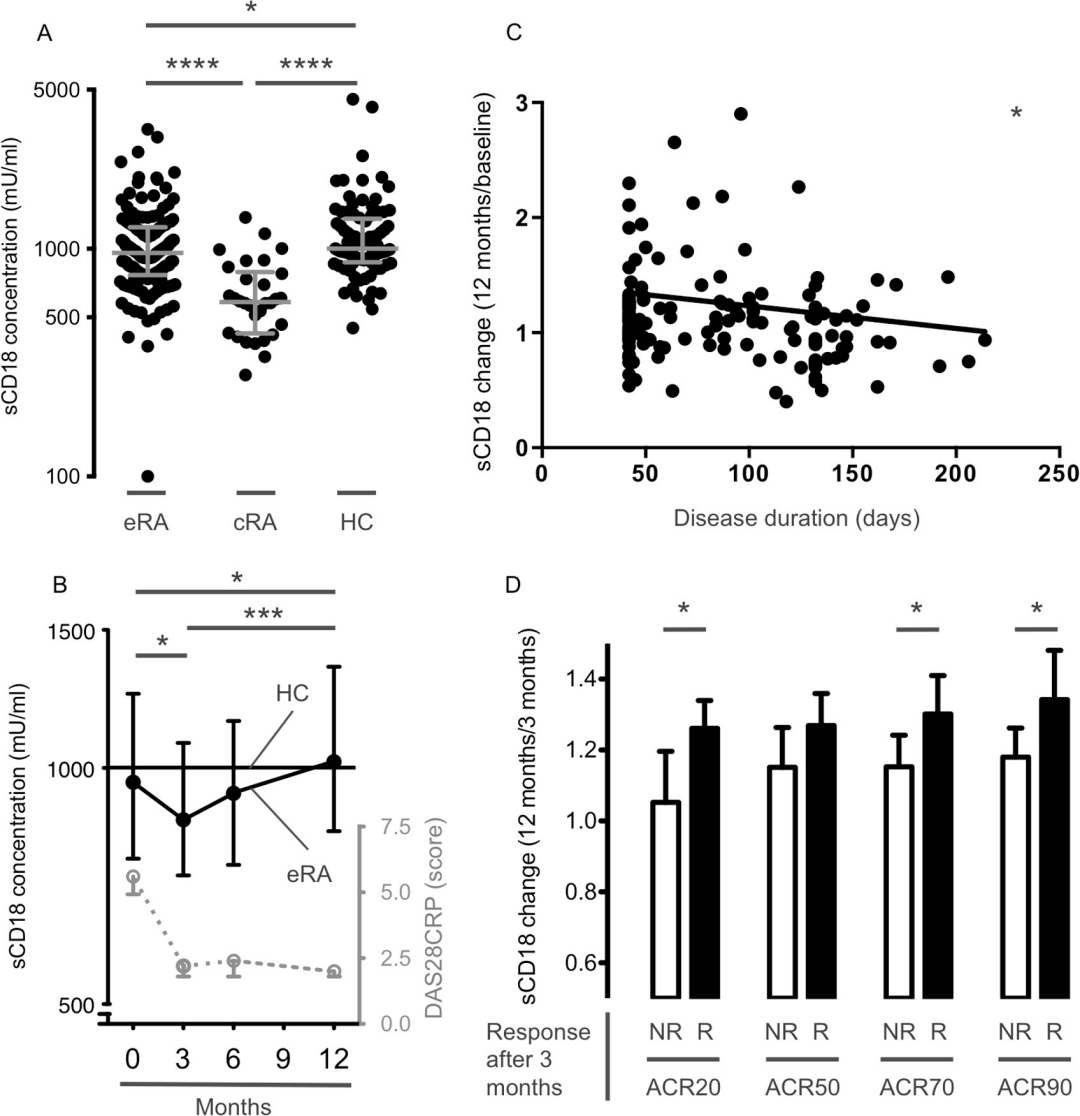
Plasma levels of sCD18 in 152 early treatment naïve RA (eRA) patients during 12 months of treatment, 30 chronic RA (cRA) patients, and 88 healthy controls (HC). **(A)** Plasma levels of sCD18 in early RA patients at the time of inclusion, in chronic RA patients, and in HCs. Lines indicate median and whiskers indicate IQR. Data were analyzed using the student’s t-test on log-transformed data. **(B)** Plasma levels of sCD18 in RA patients during 12 months of treatment. Symbols and lines indicate median and IQR. DAS28CRP score serves as a measure of clinical disease (symbols and lines indicate median and IQR). Data were analyzed with the paired t-test on log-transformed data. (**C**) Association between ratio of the change in plasma sCD18 levels from baseline to 12 months after treatment and disease duration. Data were analyzed using the Spearman correlation. **(D)** Ratio of the change in plasma sCD18 levels from 3 to 12 months after treatment in ACR non-responders (NR) and ACR responders (R) after 3 months of treatment. The mean increase in plasma levels of sCD18 from 3 to 12 months after treatment was greater in ACR responders compared with ACR non-responders. Boxes and error bars indicate mean and 95% CI. Data were analyzed using the student’s t-test. Months indicate time after inclusion (treatment initiation). * *P* < 0.05, *** *P* < 0.001, and **** *P* < 0.0001.

Plasma samples from early RA, chronic RA patients and HCs were used to establish differences depending on disease duration. Plasma samples from patients with early RA before and during a treat-to-target treatment strategy (samples from the OPERA cohort) were used to describe longitudinal changes in systemic sCD18 levels. The levels of sCD18 in plasma from patients with chronic RA were significantly lower than in plasma from both early RA patients and HCs (both *P*<0.0001). Although the levels of sCD18 in plasma from early RA patients were significantly lower than in plasma from HCs (*P*<0.05) a clear overlap was seen between the two groups (Fig 1A). The sCD18 levels exhibited a biphasic course during a treat-to-target strategy with an initial decline (*P*<0.05) followed by a gradual increase to HC levels (*P*<0.005) (Fig 1B).

### The increase in plasma sCD18 levels was greater in patients with very early disease rapidly achieving an ACR response

We further analyzed associations between the changes in sCD18 levels and disease duration and clinical treatment response in the early RA patients. The increase in plasma sCD18 levels from baseline to 12 months of treatment was inversely associated with disease duration (ρ = 0.19, P<0.05) (Fig 1C). Further, patients achieving an ACR20, ACR70, and ACR90 response after 3 months had a greater increase in plasma sCD18 levels in the subsequent time period from 3 to 12 months of treatment (all *P*<0.05) (Fig 1D). Thus, ACR20 non-responders had no increase in plasma sCD18 levels (mean ratio 1.05 (95% CI 0.909–1.20)) while ACR90 responders had the greatest increase in plasma sCD18 levels (mean ratio 1.34 (95% CI 1.20–1.48)). The changes in plasma sCD18 level did not differ between patients randomized to methotrexate in combination with adalimumab and patients receiving methotrexate in combination with placebo (S1 Fig).

### The plasma levels of sCD18 associated inversely with joint space narrowing

Based on the measurements made above, potential associations were analyzed between the plasma sCD18 levels and disease activity scores. The levels of plasma sCD18 after 3, 6 and 12 months of treatment were associated with progression in radiographic joint space narrowing after 12 months (Table 2), and the change in plasma sCD18 during the first 12 months of treatment tended to correlate with radiographic progression the subsequent 12 months (S1 Table). There were no significant associations between either baseline plasma sCD18 levels or changes in plasma sCD18 levels and age, gender, RF positivity, anti-CCP positivity, patient global, physician global, HAQ or DAS28CRP (S2 Table).

**Table 2:** Correlations of sCD18 with radiographic progression.

Radiographic progression		(Score_12 months_−Score_Baseline_)		
sCD18		TSS	JSN	Erosions
0 months	ρ	-0.09	-0.09	-0.06
	*P*	0.27	0.27	0.46
3 months	ρ	**-0.26**	**-0.28**	-0.11
	*P*	**0.0030**	**0.0013**	0.23
6 months	ρ	**-0.19**	**-0.20**	-0.12
	*P*	**0.026**	**0.021**	0.17
12 months	ρ	-0.12	**-0.18**	-0.042
	*P*	0.19	**0.034**	0.63

Data were analyzed using the Spearman correlation. ρ, Spearman’s rho. Months indicate time after inclusion (treatment initiation). Radiographic progression is measured as the difference in TSS, total Sharp score. JSN, joint space narrowing.

### The serum levels of sCD18 were lower in mice with established autoimmune arthritis and exhibited a biphasic course during arthritis development

**Fig 2:**
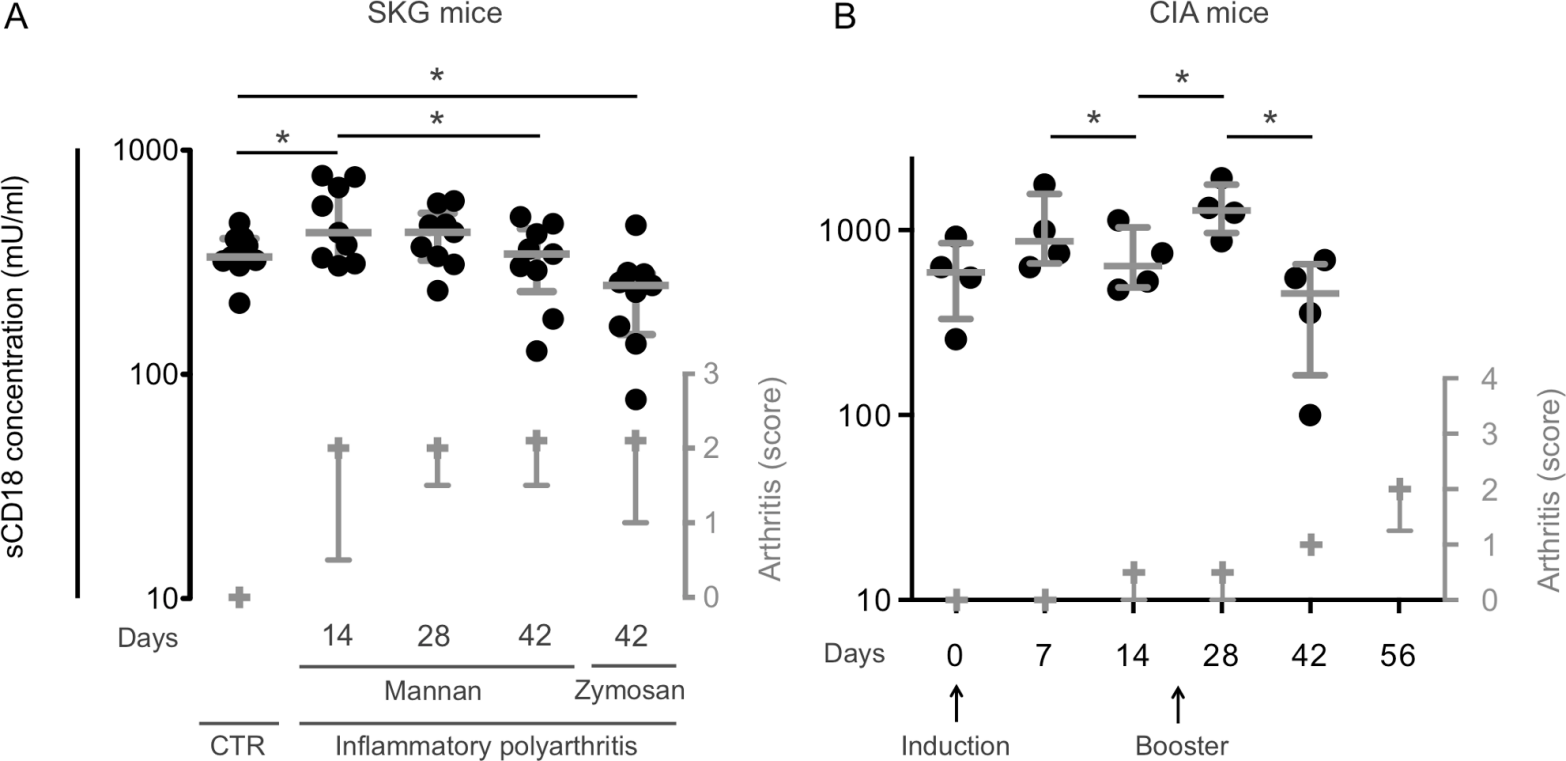
Serum levels of sCD18 in SKG and CIA mice. **(A)** The median value of serum sCD18 in healthy SKG mice was lower compared with mice 14 days after arthritis induction with mannan but higher compared with mice 42 days after arthritis induction with zymosan. n = 9. **(B)** The median value of serum sCD18 in CIA mice was increased after each collagen injection followed by a secondary decrease. n = 4. Lines indicate median and whiskers indicate IQR. Data were analyzed using the student’s t-test on log-transformed data. Arthritis score serves as a measure of clinical disease (symbols and lines indicate the median and IQR). * *P* < 0.05.

We used two murine models of rheumatoid arthritis (the SKG model and the CIA model) to assess the changes in systemic sCD18 levels before and during arthritis induction. The serum level of sCD18 showed a biphasic course after arthritis induction with mannan with an initial increase and a secondary decline and was decreased in SKG mice 42 days after arthritis induction with zymosan compared with control SKG mice without arthritis (all *P*<0.05) (Fig 2A). There was no significant difference between SKG mice 42 days after arthritis induction with mannan compared with control SKG mice without arthritis. Following the induction of CIA the serum levels of sCD18 showed an initial increase after each injection of collagen with a secondary decline (*P*<0.05) (Fig 2B).

### The in vitro CD18 shedding was increased by TNFα and decreased by adalimumab

**Fig 3:**
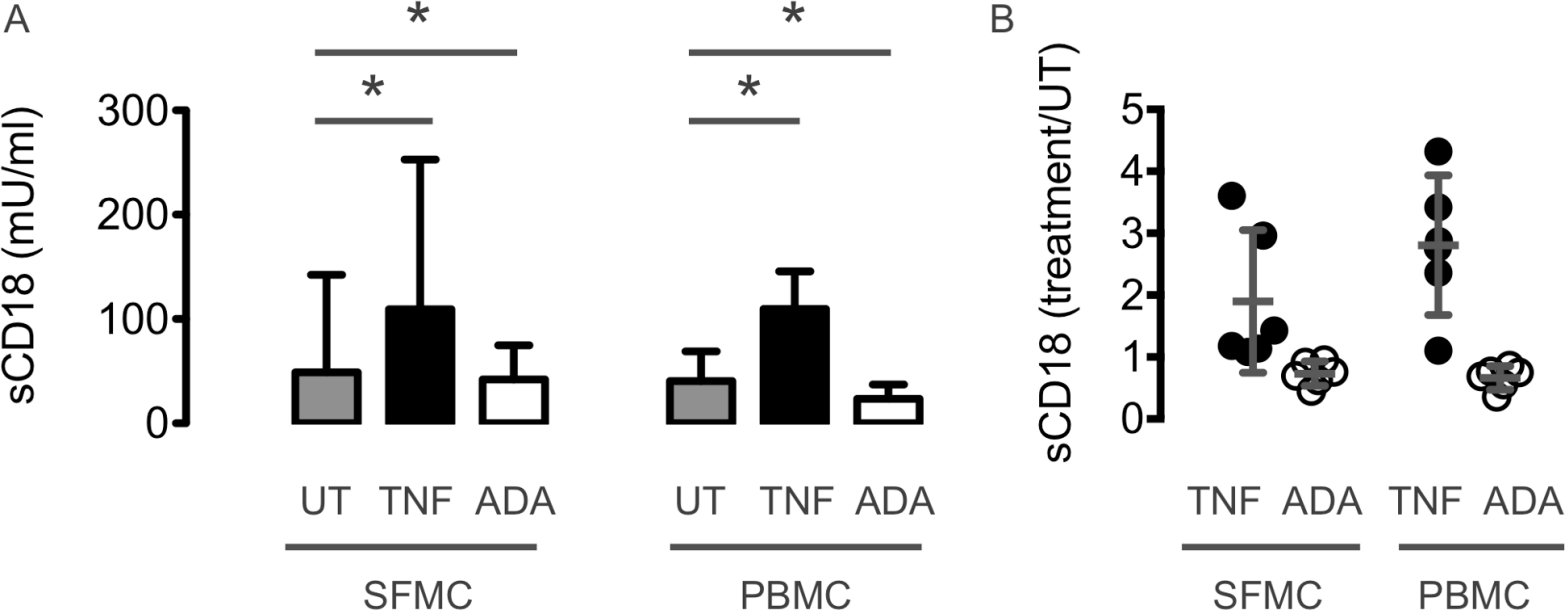
In vitro CD18 shedding from SFMC and PBMC from 6 chronic RA patients with disease flare after stimulation with TNFα or inhibition with adalimumab (ADA). The concentration of sCD18 from RA SFMC and PBMC was increased by TNFα and decreased by adalimumab compared with untreated cultures (UT). n = 6. **(A)** The concentration of sCD18 measured as mU/ml. **(B)** The relative change in sCD18 compared with untreated cultures. Boxes and error bars indicate median and interquartile range. Data were analyzed using non-parametric statistics. * *P* < 0.05.

In vitro cell cultures with SFMC and PBMC from chronic RA patients with disease flare were used to study the regulation of CD18 shedding from leukocytes. Shedding of CD18 from RA SFMC and PBMC was increased by adding recombinant human TNFα and decreased by neutralizing TNFα with adalimumab (all *P*<0.05) (Fig 3A and 3B).

## Discussion

In early RA, clinical disease characterized by swollen and painful joints is caused by synovitis. However, presence of autoantibodies may precede the clinical onset of RA by several years, and joint damage can progress despite clinical remission. In this way, clinical disease can be seen as the “tip of the iceberg” with immune activation even in patients without symptoms. Therefore, early and aggressive synovitis suppression has become the principal goal in “treat-to-target” strategies. However, the temporal changes of immune system abnormalities during disease development and therapy and their significance remain poorly understood.

In our previous studies, plasma sCD18 levels were decreased in chronic RA and SpA patients [12,19]. The circulating sCD18 is shed by leukocytes and can bind ICAM-1. Thus, the plasma concentration of sCD18 is a result of a balance between production by sheddase activity and depletion by ligand binding. In this way, decreased levels of plasma sCD18 in chronic arthritis seems to result from insufficient shedding of CD18 from monocytes and binding of sCD18 to ICAM-1 [19,27,28]. The findings in this study support that changes in systemic sCD18 accompanying clinical disease occur in biphasic patterns.

The initial phase of arthritis immediately after induction seems to be characterized by increased sCD18 levels. Thus, there was an initial increase in serum sCD18 levels 14 days after arthritis induction in the SKG mice. Also, the serum levels of sCD18 increased immediately after collagen injection in the CIA mice. This initial phase of arthritis induction in SKG and CIA mice could be simulated in vitro. The pro-inflammatory cytokine TNFα thus increased the concentration of sCD18 in both SFMC and PBMC cultures.

The later phase of arthritis with established and chronic disease seems to be characterized by progressively decreasing sCD18 levels. Thus, plasma sCD18 levels were markedly decreased in chronic RA patients compared with both early RA patients and HCs, while the decrease in plasma sCD18 levels observed in early RA patients compared with HCs was less pronounced. Accordingly, in the SKG mice the serum levels of sCD18 were decreased after 42 days and in the CIA mice the increase in serum levels of sCD18 after collagen injection were followed by a secondary decrease. This indicates that decreased levels of sCD18 reflect inflammation in transition from early to chronic disease.

The decreased plasma sCD18 levels in patients with early RA could be normalized during a treat-to-target strategy showing a biphasic pattern. There was an initial decrease after 3 months of treatment followed by a gradual increase to HC levels after 12 months of treatment. The initial phase of treatment could be simulated in vitro. Anti-inflammatory treatment with adalimumab thus decreased the concentration of sCD18 in both SFMC and PBMC cultures. The late increase in sCD18 was particularly pronounced in patients with very early disease who quickly achieved an ACR response. This suggests that immune system abnormalities are only restored in the early phase of disease and after several months of clinical remission. We speculate that the secondary increase in systemic sCD18 during remission is caused by a slowly adjusted equilibrium between production and depletion. This is supported by previous studies showing changes in monocyte subsets and suppression of endothelial cell activity during treatment of RA [19,27,28].

Our findings support that changes in systemic sCD18 accompanying clinical disease could be important for future radiographic progression in early RA patients. The plasma levels of sCD18 were only associated with JSN and not bone erosions. This is surprising as the two processes are often connected. However, the two radiographic measures are not always coupled as demonstrated by the protective effect of the anti-receptor activator of nuclear factorκB ligand denosumab on bone erosion and not JSN.[29] JSN is thus believed to be caused by the degradation of surface cartilage by matrix metalloproteinases from fibroblast like synovial cells and myeloid cells rather than osteoclasts. In this way, infiltration of myeloid cells could be the link between low sCD18 plasma levels resulting in low ICAM-1 buffer capacity and progression of JSN.

Notably the biphasic changes in systemic sCD18 accompanying clinical disease occur with latency. Thus, there is an unmet need for markers that can guide withdrawal of anti-TNFα treatment in patients with RA [30]. Using normalization of plasma sCD18 as a marker of immune system restoration could potentially be helpful. Thus, this would enable anti-TNFα withdrawal at the time of immune system remission and not just guided by longstanding clinical remission. However, further studies are needed to elucidate whether sCD18 has a role as a marker of immunological changes.

It is also possible that a recombinant form of sCD18 could be used as a drug to inhibit inflammation in RA. Currently, there are no available drugs targeting the interaction between β2 integrins and their ligands. The monoclonal antibody against ICAM-1 (enlimumab) could not be administered repeatedly because of its mouse origin.[31], and the monoclonal antibody blocking LFA-1 (efalizumab) had serious side effects due to its strong immunosuppressive actions [32]. In this context, sCD18 appear to be a possible inhibitor because it is found in both health and disease and does not block interaction completely. Investigations with LFA-1 small-molecule antagonists (e.g. BMS-688521) or endogenous antagonists (e.g. Del-1) to treat autoimmune disease are ongoing [33–35].

One shortcoming of the animal data is that they do not necessarily reflect human disease. We used SKG and CIA mice because we did not have plasma samples from the RA patients before they developed clinical disease. The SKG and CIA mouse models seem to be feasible for studying immune system alterations before and during arthritis development. The SKG and collagen-induced arthritis (CIA) mouse model of arthritis share important features with human RA. SKG mice present with symmetric involvement of small joints, elevated serum levels of pro-inflammatory cytokines, presence of rheumatoid factor and joint destruction [36]. Mouse CIA is characterised by symmetrical joint involvement with synovitis, pannus formation and cartilage and bone erosion [37]. Another weakness is that the difference in serum sCD18 levels between SKG mice with arthritis and SKG mice without arthritis only reached significance when using zymosan as arthritis inducer, despite that there was no difference in arthritis score between the mannan and Zymosan groups [22]. Another obvious weakness of potentially using systemic sCD18 as a marker of immunological changes is the rather small changes observed.

## Conclusion

The plasma sCD18 levels were altered in patients with early RA, in mice with autoimmune arthritis and in cell cultures treated with TNFα and adalimumab. Although the changes in circulating sCD18 reported here could represent temporal immunological changes during treatment of RA and arthritis induction in animals, the clinical utility of sCD18 as a biomarker of treatment response and disease progression seems to be limited due to the numerically small oscillations.

## Supporting Information

S1 Fig The changes in plasma sCD18 in patients randomized to methotrexate in combination with adalimumab (MTX+ada) and patients receiving methotrexate in combination with placebo (MTX+pla). Symbols indicates median. Line indicate HC median.
(TIFF)

S1 Table Associations between changes in sCD18 and radiographic progression. Data were analyzed using the Spearman correlation. ρ, Spearman’s rho. Months indicate time after inclusion (treatment initiation). TSS, total Sharp score. JSN, joint space narrowing.
(DOCX)

S2 Table Associations between baseline plasma sCD18 levels and changes in plasma sCD18 levels and age, gender, RF positivity, anti-CCP positivity, patient global, physician global, HAQ or DAS28CRP. Data were analyzed using the Spearman correlation. Spearman’s ρ and *P* value in parenthesis.
(DOCX)

## Abbreviations

ACR: American College of Rheumatology improvement score
CCP: cyclic citrullinated peptide
CD: cluster of differentiation
CIA: collagen-induced arthritis
CRP: C-reactive protein
DAS: disease activity score
EDTA: ethylenediamine tetraacetic acid
FCS: fetal calf serum
HAQ: health assessment questionnaire
HC: healthy control
ICAM-1: intercellular adhesion molecule 1
Ig: immunoglobulin
IQR: interquartile range
LFA-1: lymphocyte function-associated antigen 1
Mac-1: macrophage-1 antigen
OPERA: OPtimized treatment algorithm in Early Rheumatoid Arthritis
PBMC: peripheral blood mononuclear cell
PBS: phosphate buffered saline
R: receptor
RA: rheumatoid arthritis
RF: rheumatoid factor
S: soluble
SFMC: synovial fluid mononuclear cell
TBS: tris buffered saline
TNFα: tumor necrosis factor alpha
TRIFMA: time-resolved immunfluorometric assay
UT: untreated
