# Supplementary figures and images for: Changes in Soluble CD18 in Murine Autoimmune Arthritis and Rheumatoid Arthritis Reflect Disease Establishment and Treatment Response
Source: PLoS One. 2016 Feb 5;11(2):e0148486. doi: 10.1371/journal.pone.0148486 (PMC4743942; doi:10.1371/journal.pone.0148486)

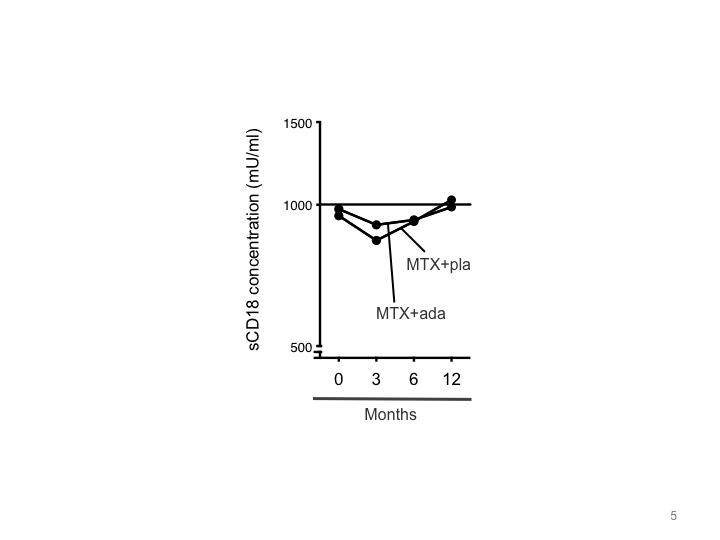

Supplement: S1 Fig — Symbols indicates median. Line indicate HC median. (TIFF) [file pone.0148486.s001.tiff]
