# Supplementary material for: Changes in Soluble CD18 in Murine Autoimmune Arthritis and Rheumatoid Arthritis Reflect Disease Establishment and Treatment Response
Source: PLoS One. 2016 Feb 5;11(2):e0148486. doi: 10.1371/journal.pone.0148486 (PMC4743942; doi:10.1371/journal.pone.0148486)
Supplement: S1 Table — Data were analyzed using the Spearman correlation. ρ, Spearman’s rho. Months indicate time after inclusion (treatment initiation). TSS, total Sharp score. JSN, joint space narrowing. (DOCX) [file pone.0148486.s002.docx]

| Radiographic progression |  | Score (24 months–12 months) | | |
| --- | --- | --- | --- | --- |
| sCD18 change |  | TSS | JSN | Erosions |
| sCD18 (12 months/baseline) | ρ | -0.10 | -0.17 | 0.01 |
|  | *P* | 0.28 | 0.055 | 0.90 |
