# Supplementary material for: Changes in Soluble CD18 in Murine Autoimmune Arthritis and Rheumatoid Arthritis Reflect Disease Establishment and Treatment Response
Source: PLoS One. 2016 Feb 5;11(2):e0148486. doi: 10.1371/journal.pone.0148486 (PMC4743942; doi:10.1371/journal.pone.0148486)
Supplement: S2 Table — Data were analyzed using the Spearman correlation. Spearman’s ρ and P value in parenthesis. (DOCX) [file pone.0148486.s003.docx]

|  | **sCD18** | |
| --- | --- | --- |
|  | **Baseline** | **Change (12 months/baseline)** |
| **Baseline characteristics** |  |  |
| **Age** | 0.14 (0.082) | 0.018 (0.84) |
| **Gender** | 0.022 (0.79) | 0.038 (0.66) |
| **RF positivity** | 0.14 (0.098) | -0.12 (0.17) |
| **Anti-CCP positivity** | 0.060 (0.46) | 0.002 (0.98) |
| **Disease activity scores^a^** |  |  |
| **Patient global** | -0.080 (0.33) | -0.017 (0.84) |
| **Physician global** | -0.021 (0.80) | 0.025 (0.77) |
| **HAQ** | -0.12 (0.15) | 0.065 (0.45) |
| **DAS28CRP** | -0.061 (0.46) | -0.014 (0.87) |
|  |  |  |
